# Supplementary material for: NiCo2S4/MoS2 Nanocomposites for Long-Life High-Performance Hybrid Supercapacitors
Source: Nanomaterials (Basel). 2023 Feb 10;13(4):689. doi: 10.3390/nano13040689 (PMC9960812; doi:10.3390/nano13040689)
Supplement: Supplementary file 1 [file nanomaterials-13-00689-s001.zip › nanomaterials-2167523-supplementary.pdf]

nanomaterials

MDPI

Supplementary Materials

$NiCo_2S_4/MoS_2$ Nanocomposites for Long-life High-Performance Hybrid Supercapacitors

Le Nhu Minh Tue [1,†], Sumanta Sahoo [1,†], Ganesh Dhakal [1], Van Hoa Nguyen [2], Jintae Lee [1], Yong Rok Lee [1] and Jae-Jin Shim [1,*]

[1] School of Chemical Engineering, Yeungnam University, 280 Daehak-ro, Gyeongsan, Gyeongbuk 38541, Republic of Korea
[2] Department of Chemistry, Nha Trang University, 2 Nguyen Dinh Chieu, Nha Trang, Vietnam
* Correspondence: jjshim@yu.ac.kr; Tel.: +82-53-810-2587
† These authors contributed equally to this work.

Experimental Section

Materials

Nickel (II) nitrate hexahydrate ($Ni(NO_3)_2.6H_2O$), cobalt (II) nitrate hexahydrate ($Co(NO_3)_2.6H_2O$), and thioacetamide (C_2H_5NS) were purchased from Alfa Aesar. Sodium molybdate dihydrate ($Na_2MoO_4.2H_20$), ethylenediamine ($C_2H_8N_2$), and ethylene glycol ($C_2H_6O_2$) were obtained from Sigma-Aldrich, Alfa Aesar, and Daejung, respectively.

Materials Characterizations

NCMS-L, NCMS-H, $NiCo_2S_4$, and MoS_2 were analyzed structurally by X-ray diffraction (XRD, PANalytical, X'Pert-PRO MPD) using Cu Kα radiation (0.154 nm), X-ray photoelectron spectroscopy (XPS, Thermo Scientific, K-Alpha) using Al Kα monochromatized radiation, field-emission scanning electron microscopy (FE-SEM, HITACHI, S-4800), transmission electron microscopy (TEM, Philips, CM-200) at an acceleration voltage of 200 kV, and high-resolution transmission electron microscopy (HR-TEM, TITAN G2 ChemiSTEM) using a Cs Probe electron microscope.

Electrochemical measurements

The electrochemical performance was measured by EIS (electrochemical impedance spectroscopy), CV (cyclic voltammetry), and GCD (galvanostatic charge/discharge) using an electrochemical workstation potentiostat/galvanostat (Metrohm, AutoLab PGSTAT 302N) with a three-electrode system in 3 M KOH electrolyte. A working electrode coated with active materials on 3D nickel foam (NF), a platinum counter electrode, and a saturated calomel reference electrode (SCE) was used as the working, counter, and reference electrodes in the three-electrode system. The working electrode was prepared by mixing the active material, carbon black, and polymer binder (Nafion) at a weight ratio of 80:15:5. The slurry was pressed onto NF and dried at 60°C for 10 h. Moreover, a hybrid supercapacitor device was fabricated with NCMS-L as the positive and activated carbon (AC) as the negative electrode. A cellulose filter paper was used as the separator and placed between the positive and negative electrodes.

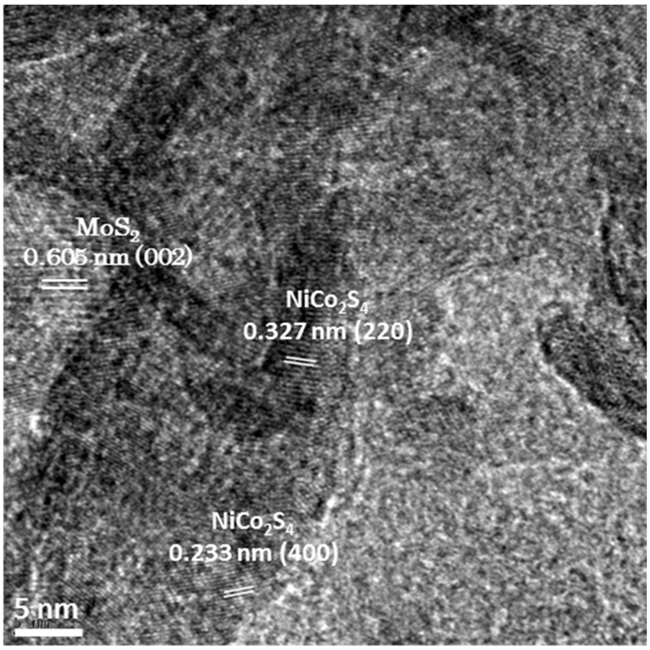

Figure S1. HRTEM image of NCMS-L sample.

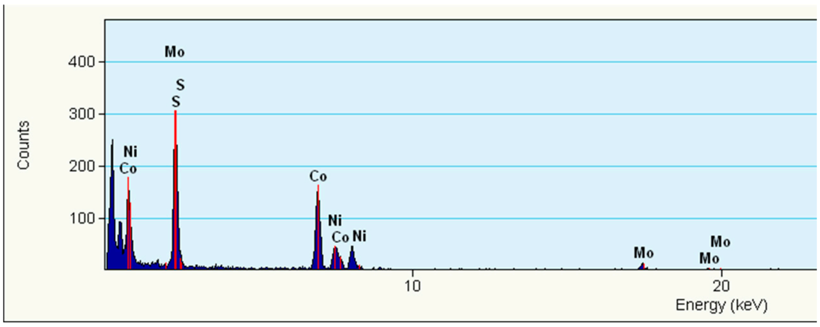

Figure S2. EDX of NCMS-L sample.

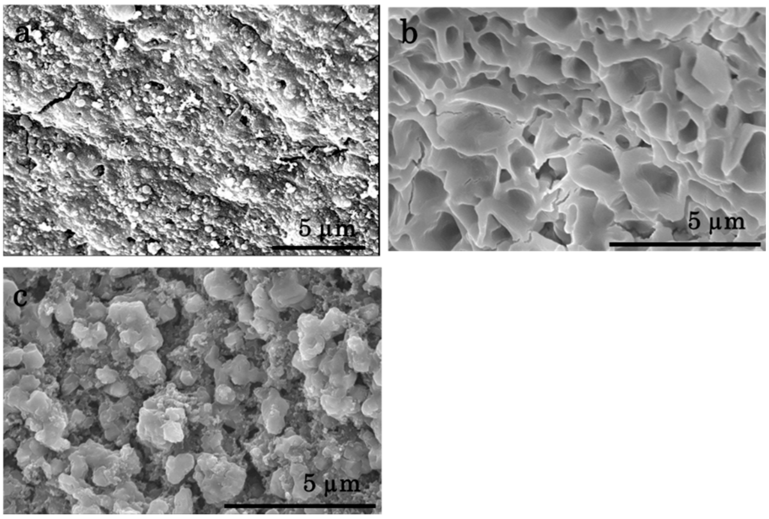

Figure S3. SEM images of (a) NCMS-L after 45000 cycles and of (b) MoS_2 and (c) $NiCo_2S_4$ after 5000 cycles.

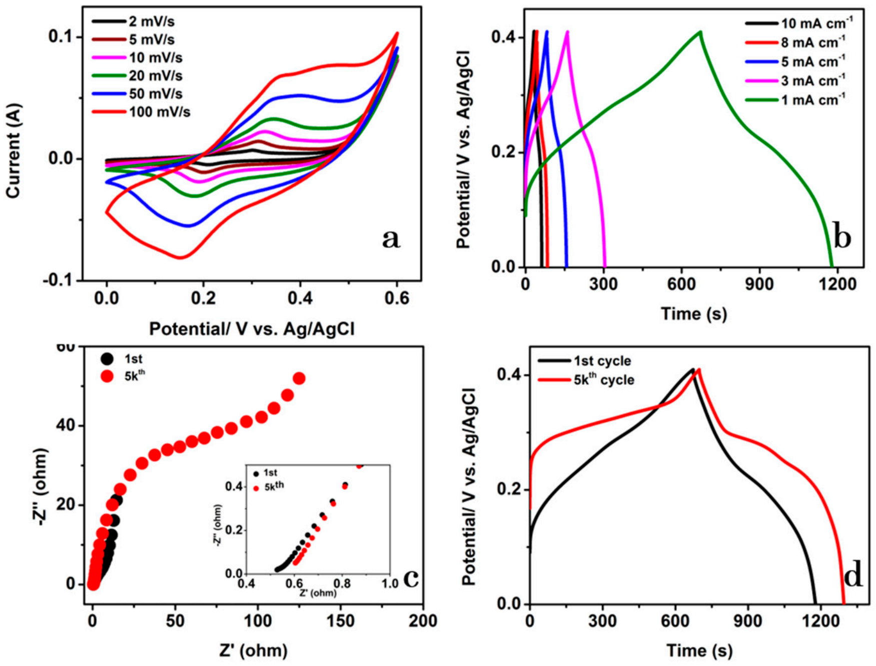

Figure S4. Electrochemical properties of $NiCo_2S_4$: (a) CV curves recorded at various scan rates, (b) GCD curves at different currents from 1 mA to 10 mA with current densities of 0.8, 2.3, 3.8, 6.2, and 7.7 A g^{-1}, respectively, (c) Nyquist plots in the frequency range from 0.01 Hz to 100 kHz and (d) GCD curve of the first cycle and after 5000 cycles with the specific capacitance increasing from 947 to 1121 F g^{-1} at a current of 1 mA (current density 0.8 A g^{-1}).

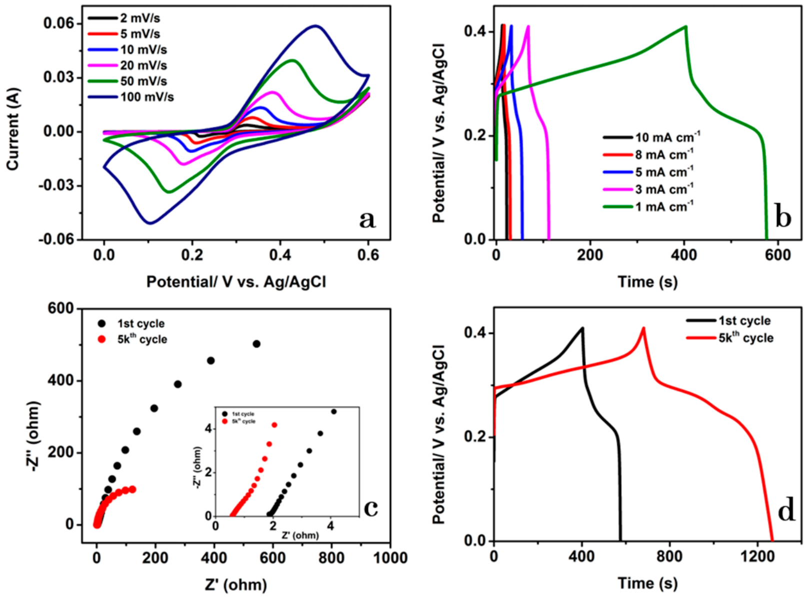

Figure S5. Electrochemical properties of MoS_2: (a) CV curves recorded at various scan rates, (b) GCD curves at different currents from 1 mA to 10 mA with a current density 0.8, 2.3, 3.8, 6.2, and 7.7 A g^{-1}, respectively, (c) Nyquist plots within the frequency range from 0.01 Hz to 100 kHz and (d) GCD curve of the first cycle and after 5000 cycles with a specific capacitance increasing from 323 to 1100 F g^{-1} at a current of 1 mA (current density 0.8 A g^{-1}).

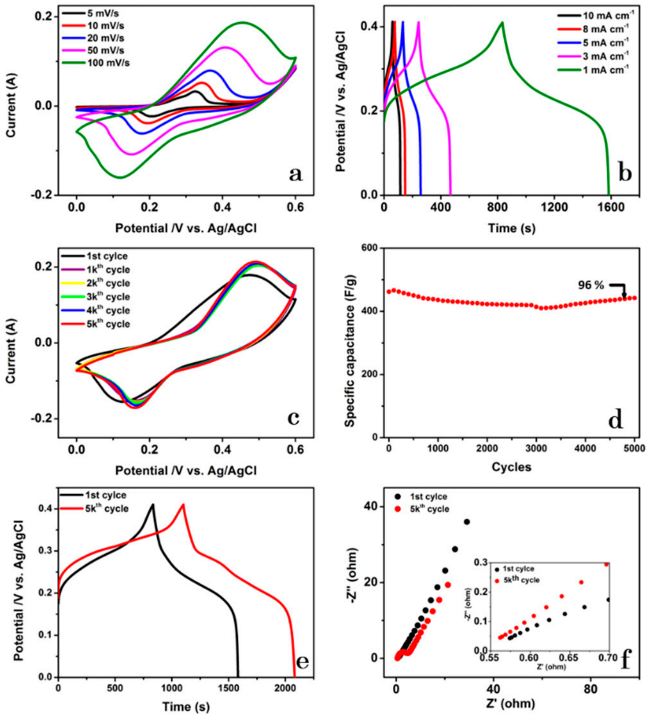

Figure S6. Electrochemical properties of NCMS-H: (a) CV curves recorded at various scan rates, (b) GCD curves at different currents from 1 mA to 10 mA with a current density 0.8, 2.3, 3.8, 6.2, and 7.7 A g^{-1}, respectively, (c) CV curves at various cycles with a scan rate of 100 mV s^{-1}, (d) Retention during 15000 cycles by CV, (e) GCD curves at the first cycle and 5000 cycles with a specific capacitance of 1405 F g^{-1} and 1840 F g^{-1} at a current of 1 mA, respectively, (f) Nyquist plots within the frequency range from 0.01 Hz to 100 kHz.

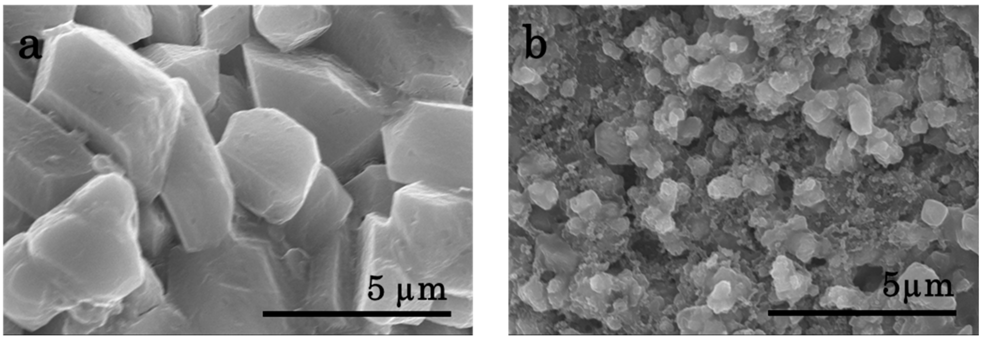

Figure S7. (a, b) SEM images of (a) MoS_2 and (b) $NiCo_2S_4$ after 5000 cycles and two weeks' pause.

Table S1. Comparison of the specific capacitance, energy density, power density, and stability of the $NiCo_2S_4/MoS_2$//AC devices with other related devices.

Electrode Material	Specific Capacitance	Energy Density	Power Density	Potential Window	Cycling Stability	Ref.
$NiCo_2S_4$ nanoparticles // AC (Device)	~ 90.5 F g^{-1}	28.3 Wh kg^{-1}	9.8 kW kg^{-1}	0–1.5 V	91.7% 5000 cycle	S1
$NiCo_2S_4$ hollow MS // AC (Device)	~ 79 F g^{-1}	24.7 Wh kg^{-1}	17.12 kW kg^{-1}	0–1.5 V	105.6% 5000 cycle	S2
$NiCo_2O_4$ // AC (Device)	49.3 F g^{-1}	15.42 Wh kg^{-1}	7.8 kW kg^{-1}	0–1.5 V	106% 2500 cycle	S3
MoS_2/GO	270 F g^{-1}	37.5 Wh kg^{-1}	2.5 kW kg^{-1}	−0.8–0.2 V	89.6% 1000 cycle	S4
**$NiCo_2S_4/MoS_2$ //AC (Device)**	**90 F g^{-1}**	**31.9 Wh kg^{-1}**	**1.26 kW kg^{-1}**	**0–1.6 V**	**102% 15000 cycle**	**This work**

*AC: Activated carbon, MS: Microsphere

References

S1. Zhu, Y.; Wu, Z.; Jing, M.; Yang, X.; Song, W.; Ji, X. Mesoporous $NiCo_2S_4$ nanoparticles as high-performance electrode materials for supercapacitors. *Journal of Power Sources* **2015**, 273, 584-590.

S2. Zhu, Y.; Ji, X.; Wu, Z.; Liu, Y. $NiCo_2S_4$ hollow microsphere decorated by acetylene black for high-performance asymmetric supercapacitor. *Electrochimica Acta* **2015**, 186, 562-571.

S3. Lu, X.-F.; Wu, D.-J.; R.-Z. Li, Q. Li, S.-H. Ye, Y.-X. Tong, G.-R. Li, Hierarchical $NiCo_2O_4$ nanosheets@hollow microrod arrays for high-performance asymmetric supercapacitors. *Journal of Materials Chemistry A* **2014**, 2, 4706-4713.

S4. Thangappan, R.; Kalaiselvam, S.; Elayaperumal, A.; Jayavel, R.; Arivanandhan, M.; Karthikeyan, R.; Hayakawa, Y. Graphene decorated with MoS_2 nanosheets: a synergetic energy storage composite electrode for supercapacitor applications. *Dalton Transactions* **2016**, 45, 2637-2646.
